# Clinical utility of circulating cell-free DNA in advanced colorectal cancer

**DOI:** 10.1371/journal.pone.0183949

**Published:** 2017-08-29

**Authors:** Allan A. Lima Pereira, Maria Pia Morelli, Michael Overman, Bryan Kee, David Fogelman, Eduardo Vilar, Imad Shureiqi, Kanwal Raghav, Cathy Eng, Shanequa Manuel, Shadarra Crosby, Robert A. Wolff, Kimberly Banks, Richard Lanman, AmirAli Talasaz, Scott Kopetz, Van Morris

**Affiliations:** 1 Department of Gastrointestinal Medical Oncology, The University of Texas MD Anderson Cancer Center, Houston, Texas, United States of America; 2 Department of Clinical Cancer Prevention, The University of Texas MD Anderson Cancer Center, Houston, Texas, United States of America; 3 Guardant Health, Redwood City, California, United States of America; University of Crete, GREECE

## Abstract

**Background:**

Circulating cell-free DNA (cfDNA) isolated from the plasma of cancer patients (pts) has been shown to reflect the genomic mutation profile of the tumor. However, physician and patient assessment of clinical utility of these assays in patients with metastatic colorectal cancer (mCRC) has not been previously described.

**Methods:**

Patients were prospectively consented to a prospective genomic matching protocol (Assessment of Targeted Therapies Against Colorectal Cancer [ATTACC]), with collection of blood for cfDNA extraction and sequencing of a 54-gene panel in a CLIA-certified lab. Formalin-fixed, paraffin-embedded (FFPE) tissue from prior resections or biopsies underwent 50-gene sequencing. Results from both assays were returned to the treating physicians for patient care and clinical trial selection. Follow-up surveys of treating physicians and chart reviews assessed clinical utility.

**Results:**

128 mCRC pts were enrolled between 6/2014 and 1/2015. Results were returned in median of 13 and 26 days for cfDNA and FFPE sequencing, respectively. With cfDNA sequencing, 78% (100/128) of samples had a detectable somatic genomic alteration. 50% of cfDNA cases had potentially actionable alterations, and 60% of these could be genomically matched to at least one clinical trial in our institution. 50% (15/30) of these pts enrolled onto an identified matched trial. Physicians reported that the cfDNA testing improved the quality of care they could provide in 73% of the cases, and that 89% of pts reported greater satisfaction with the efforts to personalize experimental therapeutic agents.

**Conclusions:**

cfDNA sequencing can provide timely information on potentially actionable mutations and amplifications, thereby facilitating clinical trial enrollment and improving the perceived quality of care.

## Introduction

Outcomes for patients with metastatic colorectal cancer (mCRC) have improved substantially over the past decades due to advances in multimodality therapies, including a greater utilization of metastatic resection for patients with liver-only distant disease [1–3]. However, prolongation of survival for patients with unresectable mCRC has proven more modest, highlighting the need for new and effective treatment options. Use of biomarkers to guide therapeutic decisions is already widely accepted in clinical practice. For example, expanded testing for mutations in codons 2, 3 and 4 of *KRAS* and *NRAS* influence use of anti-EGFR monoclonal antibodies[4]. Additionally, new predictive biomarkers are under investigation. The presence of a *BRAF* V600E mutation or microsatellite instability may prompt a provider to recommend clinical trials incorporating promising targeted therapies and/or immunotherapy agents, and HER2/neu overexpression or *ERBB2* amplification of on colorectal tumors also has significant implications for forthcoming trials with anti-HER2 therapies[5].

Biomarker-related decisions based on sequencing tissue samples from invasive tissue biopsies have been essential in the continued management of mCRC. However, tissue biopsy-based genotyping has important limitations. Due to the intra- and inter-tumoral heterogeneity [6], a single biopsy may not be fully representative of the disease biology, and extensive sampling of metastatic deposits to detect the entirety of genomic profiling is unfeasible [7, 8].

Recent improvements in sequencing technologies have allowed for collection of blood specimens (“liquid biopsies”) to analyze circulating cell-free tumor DNA (cfDNA) in the plasma for the presence of biomarkers relevant to mCRC. Plasma analysis is a less invasive approach when compared to traditional needle biopsies, and therefore may be attractive to providers and patients alike. Indeed, since tumor DNA is released into the blood stream during cell turnover[9], it has been postulated that cfDNA mutation results may characterize a “real-time” mutational profile of the tumor(s) more accurately than retrospectively studied formalin-fixed, paraffin-embedded (FFPE) tissue taken from biopsies or surgeries [10]. cfDNA analysis allows for tracking of dynamic changes in tumor biology throughout an individual’s treatment course and provides insight into tumor heterogeneity.

Use of cfDNA to detect mutations has been reported to be more sensitive for tracking disease status[11] and detecting recurrence[12–14] when compared to traditional laboratory markers like CEA. It has also been studied as a tool to both monitor[15, 16] and guide targeted therapies based on potentially actionable mutations detected[10]. These advantages over FFPE, therefore, may guide enrollment for patients to clinical trials based on potentially actionable mutations or amplifications.

To our knowledge, the clinical utility and practicality of obtained clinical data from cfDNA genotyping assays for practicing oncologists, relative to traditional use of FFPE specimens, have not been described. Here we report a single-institution experience with the use of sequencing results from cfDNA and FFPE samples to guide decisions in referring patients with mCRC to matched biomarker-related clinical trials.

## Methods

### Study population

In this single institution study, physicians and physician assistants at the University of Texas-MD Anderson Cancer Center were asked to compare the use of cfDNA and FFPE tissue collections for practicality and convenience in assessing mutation profiles for patients with mCRC. We recruited participants from 6/2014 to 1/2015. During this time, the respondents answered the questionnaire based on their experiences collecting clinically relevant data for each patient who consented to the Assessment of Targeted Therapies Against Colorectal Cancer (ATTACC) protocol, designed to molecularly profile tumors of patients with refractory mCRC (NCT01196130) [17]. Patients with 5-fluorouracil refractory mCRC were eligible to provide written consent and enroll on the ATTACC protocol for collection of cfDNA from both macrodissected historic FFPE and plasma samples for concurrent sequencing. This research was conducted under the approval of the University of Texas—MD Anderson Cancer Center Institutional Review Board.

### Plasma and tissue genotyping

Patients had 20mL of blood collected for cfDNA extraction and sequencing on a 54-gene next-generation sequencing panel (S1 Table) in a CLIA-certified, CAP-accredited clinical laboratory (Guardant360, Guardant Health) for point mutations and select gene copy number amplifications. DNA obtained from formalin-fixed, paraffin-embedded (FFPE) tissue from prior resection or biopsy underwent 50-gene sequencing (Ion Torrent, Life Technology). Results from both assays were returned to the treating physicians for patient care and clinical trial selection. Actionable mutations were identified based on their potential to be targeted with an investigational therapy on an available, biomarker-matched clinical trial.

### Survey instrument

The survey instrument (S1 Fig) contained questions related to following: (1) mutations or amplification identified from the cfDNA and FFPE assays, (2) ability of the returned results to provide molecular tumor characterization and to guide treatment and enrollment in clinical trials, (3) the impact of the use of cfDNA during the patient’s participation on ATTACC program towards the quality of provided care and (4) patient satisfaction with the efforts to personalize experimental options. Surveys were sent 30 days after the patients’ enrollment onto ATTACC program. Providers replied to the survey for each patient enrolled, based on their clinical interpretation and proposed management course.

### Turnaround time

Turnaround times for both cfDNA and FFPE analyses were measured in days from the date of consent until the reporting of the final sequencing results and reported as median and interquartile range (IQR).

### Statistical analysis

All data in this observational study were summarized by descriptive statistics. A post-hoc exploratory analysis of turnaround times was performed. Shapiro-Wilk test was used to test for normality of the turnaround times, which were analyzed by applying paired t-test or Wilcoxon signed rank test, as appropriate. Significance was established as 2-sided p-value <0.05.

## Results

### Patient demographics

Between 6/2014 and 1/2015, 151 patients with mCRC were enrolled onto the ATTACC program. A total of 18 providers (10 physicians and 8 physician assistants) completed the survey and their answers regarding 128 patients are reported here. Table 1 lists the demographic information for these 128 patients.

**Table 1:** Patient demographic characteristics.

Characteristic	N = 128 (%)
Age (median/range)	53 (27–76)
Gender	
Male	65(50.8)
Female	63 (49.2)
Race/Ethnicity	
Asian	9 (7.0)
Black	13 (10.2)
Hispanic	7 (5.5)
Other	2 (1.6)
White	97 (75.8)
ECOG 0–1	128 (100)
0	19 (14.8)
1	108 (84.4)
2	1 (0.8)
Primary tumor location	
Right colon	34(26.6)
Transverse colon	5 (3.9)
Left Colon	69 (53.9)
Rectum	20 (15.6)
Microsatellite Instability	
MSS / MSI-L	110 (85.9)
MSI-H	6 (4.7)
Unknown	12 (9.4)
Systemic therapy (prior exposure)	
5-FU or Capecitabine	128 (100.0)
Irinotecan	111 (86.7)
Oxaliplatin	123 (96.1)
Anti-EGFR*	40 (31.3)
Anti-VEGF**	109 (85.2)
Regorafenib or TAS 102	16 (12.5)
Immunotherapy^♯^	1 (0.8)
Other^♯^	5 (3.9)
Sites of metastasis	
Liver	85 (66.4)
Lung	76 (59.4)
Peritoneum	31 (24.2)
Pelvis	3 (2.3)
Bone	7 (5.5)
CNS	1 (0.8)
Lymph nodes	50 (39.1)
Other	8 (6.3)
Site of collection tissue sample	
Primary tumor	82(64.1)
Metastatic site	46 (35.9)
Tissue sample location (metastatic site)	
Liver	25 (54.3)
Lung	5 (10.9)
Peritoneum/Omentum	2 (4.4)
Other	14 (30.4)

MSS Microsatellite Stable; MSI-L: Microsatellite instability-Low; MSI-H: Microsatellite instability—High; 5-FU: 5-fluorouracil; EGFR: epidermal growth factor receptor; VEGF: vascular endothelial growth factor; CNS: Central Nervous System

*—Cetuximab or panitumumab

**—Bevacizumab or aflibercept

^♯^ - Experimental treatment in prior clinical trials

### Mutation/Amplification detection

**Fig 1:**
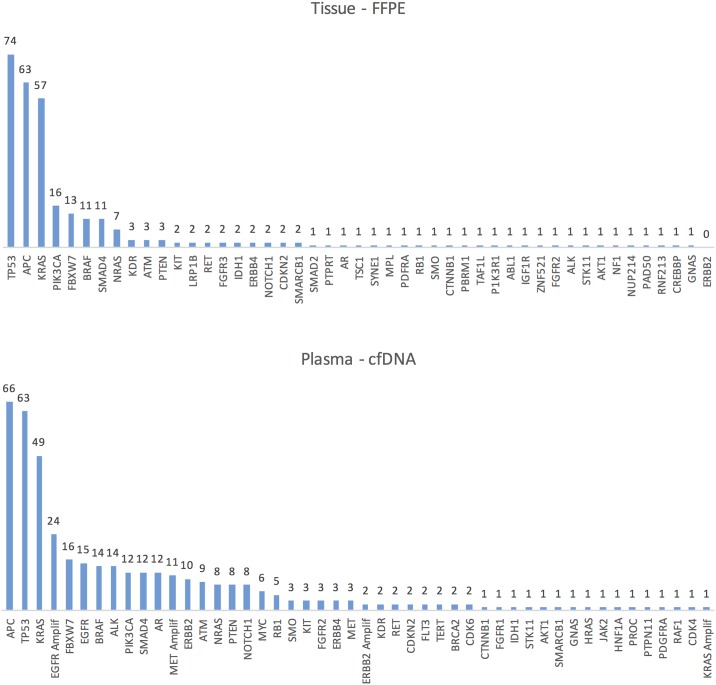
Number of patients with genomic alterations detected by both FFPE and cfDNA analysis. FFPE = formalin-fixed, paraffin-embedded tissue; cfDNA = cell-free DNA; Amplif = amplification.

Data summarizing the number of patients with a specific genomic alteration found by both cfDNA and FFPE analyses, actionable or not, are presented in Fig 1 and the absolute number of each mutation and amplification identified by cfDNA assay is presented in S2 Fig.

**Fig 2:**
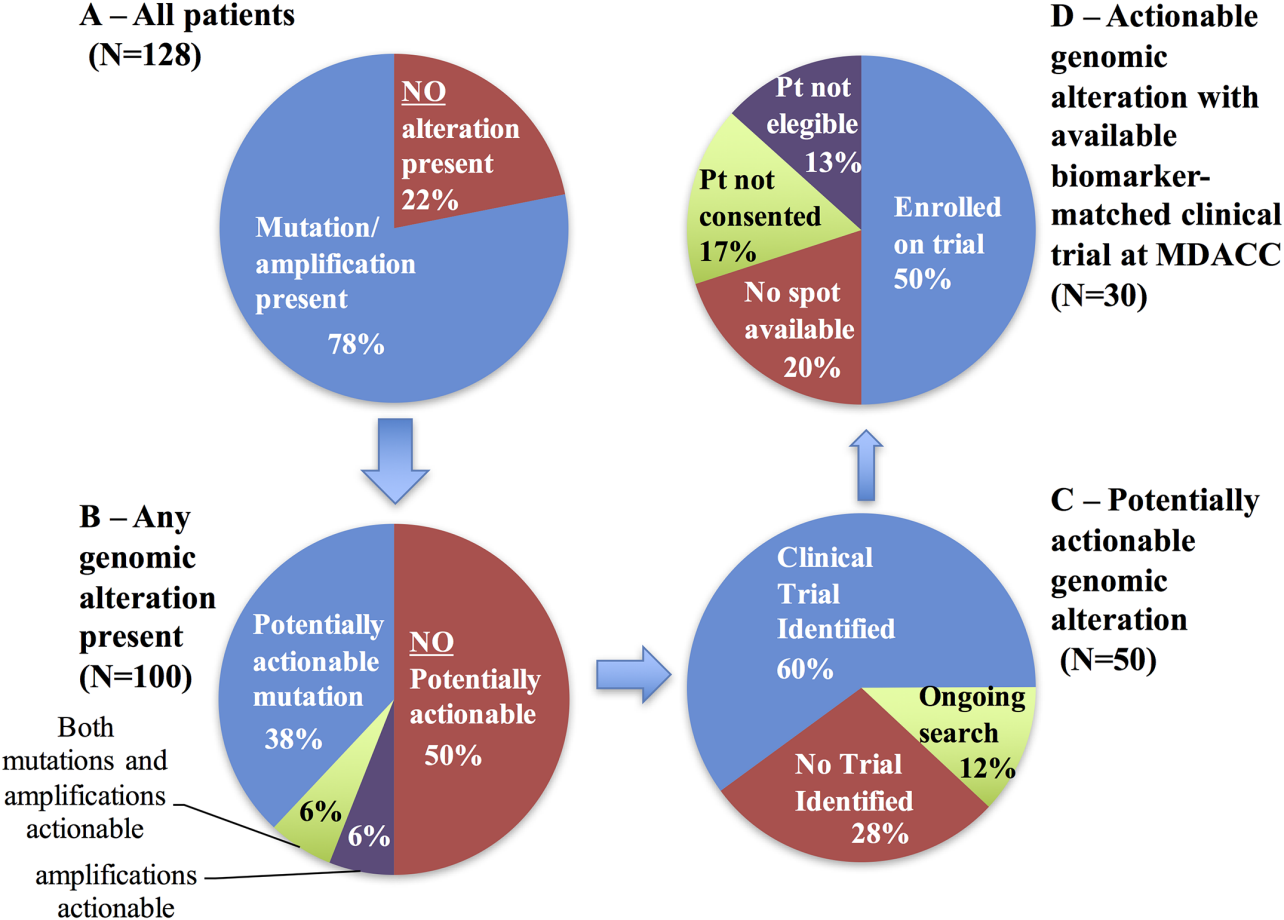
Clinical utility of cfDNA sequencing results. **(A)** Detectable mutations and/or amplification were present in 78% of patients. **(B)** 50% of these patients (N = 50) had “potentially actionable” mutations and/or amplifications. **(C)** Among these, 60% (N = 30) patients had a clinical trial identified based on the matched biomarker detected from the cfDNA (D) 15 patients ultimately enrolled on a biomarker-based clinical trial. Pt = patient; MDACC = MD Anderson Cancer Center.

As illustrated in Fig 2A, detectable mutations and/or amplification were present in cfDNA samples from 78% of patients (100/128). In 4 of the 28 cases with no detected mutation/amplification, the absence of genomic alterations was attributed to failures in sample quality. Among the 100 cases in which genomic alterations were identified by cfDNA genotyping, physicians stated that the cfDNA results were potentially actionable 50% of the time. These genomic alterations were point mutations in 38 cases, gene copy number amplifications in 6 cases, and both in the remaining 6 patients (Fig 2B).

Regarding all 128 patients included, physicians were further queried if there were any potentially actionable mutation or amplification identified by tumor tissue sequencing that was not identified by plasma sequencing. Such situations were detected in 32 patients (Table 2). While in the remainder of the cases, cfDNA provided as much clinically relevant data as matched FFPE tissue.

**Table 2:** Additional findings noted in sequencing of historic FFPE specimens compared to cfDNA sequencing. FFPE = formalin-fixed, paraffin-embedded tissue; cfDNA = cell-free DNA.

Additional findings in FFPE—Physician Survey Response	N (%)
No additional findings were present	**92 (74%)**
Additional 'potentially actionable' amplifications were present in the tissue	**13 (10%)**
Additional 'potentially actionable' mutations were present in the tissue	**16 (13%)**
Both additional 'potentially actionable' amplifications and mutations were present in the tissue	**3 (2%)**

### Enrollment onto clinical trials

Among the 50 patients in whom actionable genomic alterations were identified by cfDNA sequencing, at least one available biomarker-matched clinical trial was identified in 30 patients’ cases (60%, Fig 2C). Of these, 15 (50%, or 12% of the entire cohort of 128 patients) ultimately enrolled onto a biomarker-based clinical trial based on the cfDNA result (Fig 2D). Regarding the reasons that patients were not enrolled onto matched clinical trials, the patient did not meet eligibility criteria in 4 cases; there were no spots available in 6 cases; or the patients was not interested or did not consent for participation in 5 cases.

### Provider convenience and preference

**Fig 3:**
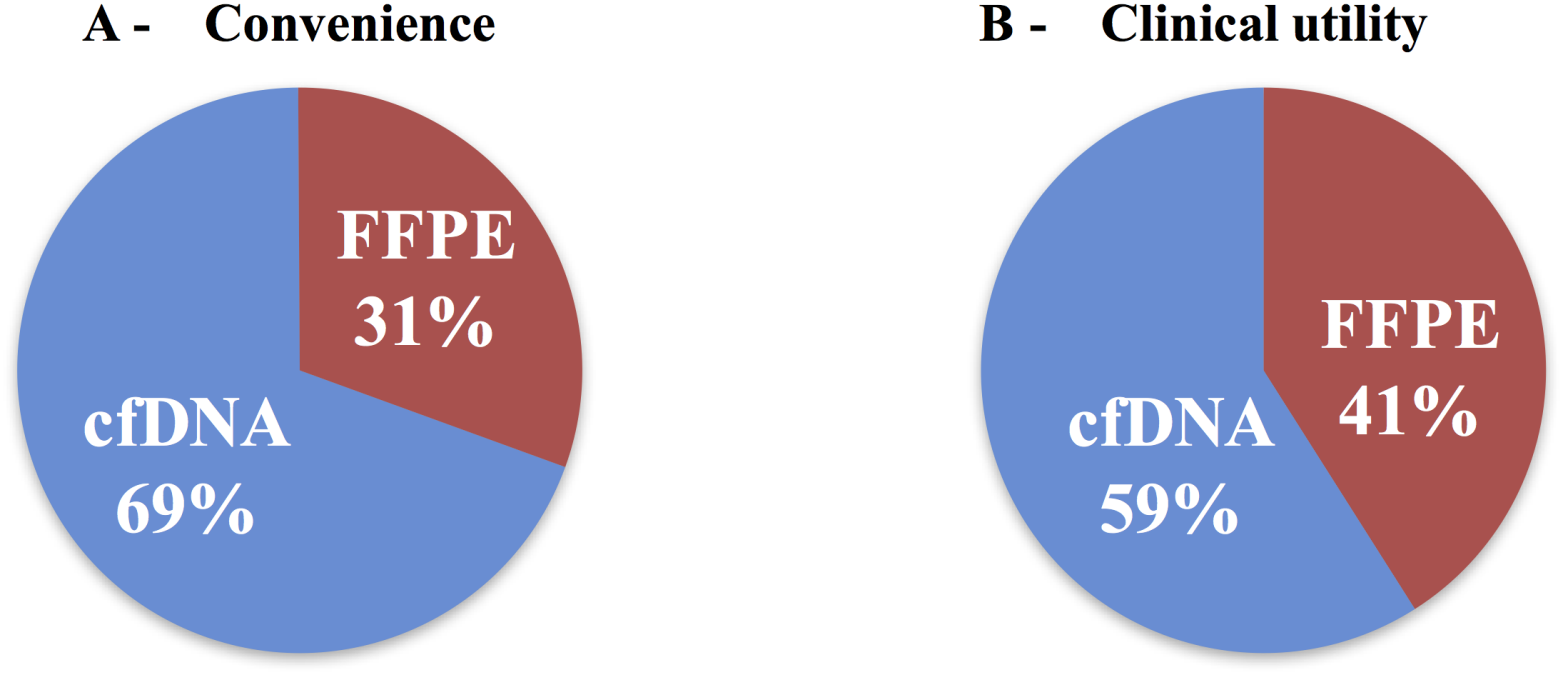
Provider survery results. Physician preference for convenience (A) and clinical utility (B) according to the sample detection method and a stated desire to incorporate sequencing results into clinical decisions. FFPE = formalin-fixed, paraffin-embedded tissue; cfDNA = cell-free DNA.

When asked about the convenience of the plasma test results compared to tissue testing, the treating providers stated that the cfDNA was more convenient than FFPE in 69% of the cases (Fig 3A). When questioned which method (cfDNA vs. FFPE) was the superior platform for each patient’s case in terms of utilizing the molecular tumor characterization to guide experimental therapy choice (“clinical utility”), cfDNA was selected by the treating physician in the majority of the cases (59 vs. 41%; Fig 3B).

### Improvement in quality of care and patient satisfaction

**Fig 4:**
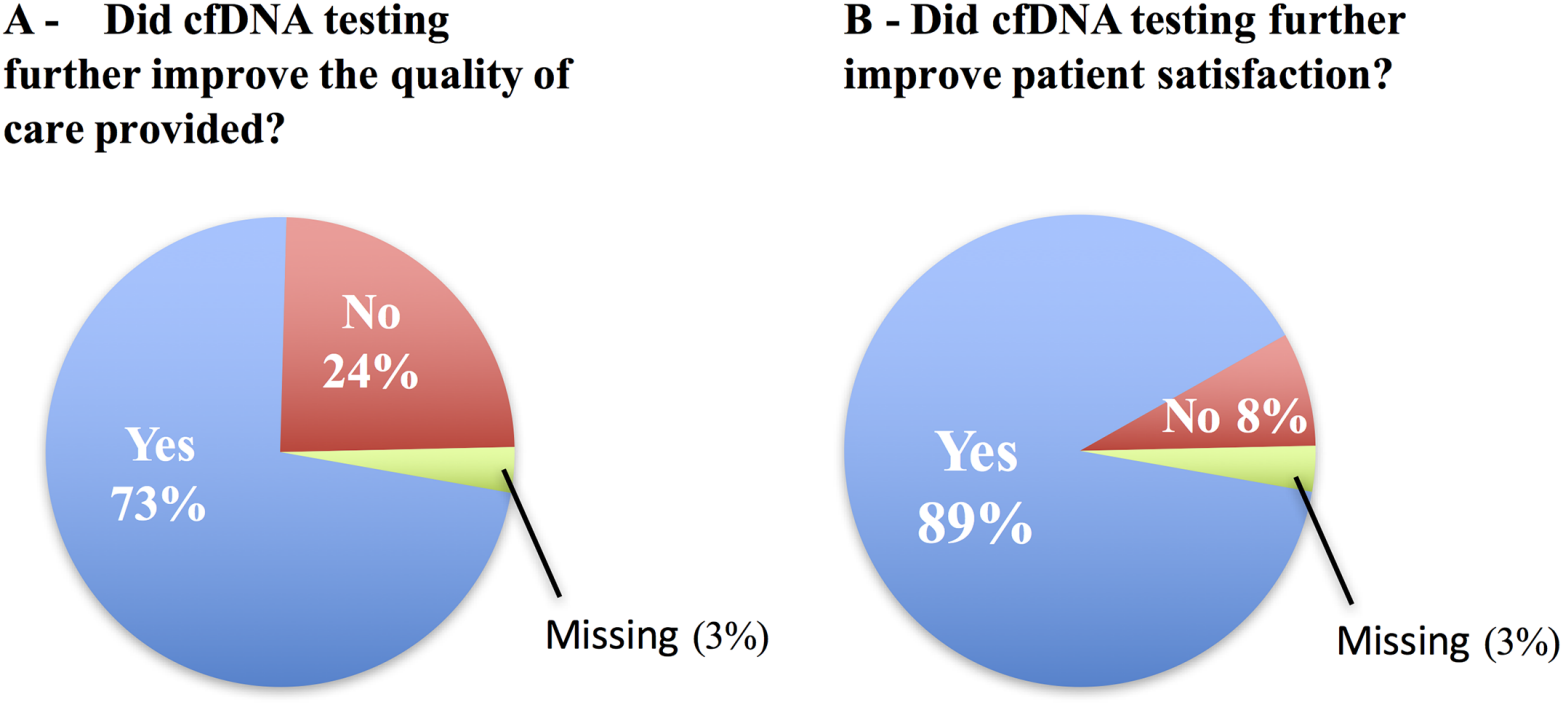
The impact of the use of cfDNA in (A) quality of care and (B) patient satisfaction.

The providers were questioned about the use of cfDNA in clinical practice and their perception of patient satisfaction with the utilization of this tool in addition to the standard tissue sequencing practice. In 73% of cases, physicians felt that cfDNA testing improved the quality of care they were able to provide, and improved patient satisfaction with the efforts to personalize experimental options in 89% of cases (Fig 4).

### Turnaround time

For cfDNA sequencing results, median turn-around time was 13 days (interquartile range [IQR], 10–15 days), approximately 50% faster than sequencing results from FFPE tissue (median 24 days, IQR 14–45 days, p<0.001). The main reason for the delay in FFPE sequencing turn-around-time resulted from the time needed to request and receive archival tissue from outside facilities for further analysis.

## Discussion

In this observational study, we retrospectively assessed the physicians’ perception of the use of genotyping data obtained by cfDNA assays and FFPE analyses for incorporation towards clinical trial selection and for clinical utility on patient care in a population of mCRC patients. In addition, improved patient satisfaction with this less invasive approach was reported in 89% of cases. Our findings collectively suggest that cfDNA sequencing can safely provide timely information on potentially actionable mutations and amplifications, thereby facilitating clinical trial enrollment and improving the perceived quality of care for patients and providers alike.

Providers preferred the use of cfDNA for molecular tumor characterization over archival FFPE tumor in almost 60% of cases, reflecting the opinion that liquid biopsies are more convenient for patients and practitioners alike. Tissue biopsies have several limitations. First, tissue samples are usually obtained by invasive procedures, with inherent risks of complications like bleeding, pain and infection, which raise cumulative risk of injuring patients when considering multiple/serial (re)biopsies over time to track dynamic changes in genomic profiles across sequential lines of therapy. Second, due to intra- and inter-tumoral heterogeneity[6], a single tumor biopsy of a single region within an otherwise geographically diverse tumor genome profile may not reflect the complete biology of the disease. This is a problem even more pronounced when analyzing archival tumor biopsies taken well before later therapies. Therapy may drive selection of resistant subpopulations, which may dominate at later points in time. Indeed, genomic profiles of primary tumors and metastases are not always concordant, and clonal evolution from this pre-existing intratumoral heterogeneity may explain this discrepancy [6, 18–20]. Therefore, a noninvasive tumor genotyping method that integrates the entirety of this genomic diversity and may also be capable of tracking clonal evolution is desirable and practicable. However, these advantages are balanced by the recognition that cfDNA from the tumor is not detectable in 22% of cases. This may be due to the limited number (54) of targeted genes in the cfDNA test as well as overall assay sensitivity. Both of these issues have been addressed in subsequent assay versions which have increased the number of targeted genes to 73, with incremental improvements in sensitivity [21, 22]. For the older 54-gene panel version studied here it is estimated that 15% of CRC patients’ tumors do not carry mutations detected with the selected assay [23]. A common reason that the level of cfDNA release is reduced below limits of detection is decreased tumor shedding due to recent chemotherapy, low tumor volume, or tumor biology [24, 25].

Several potential applications of cfDNA in clinical oncology have been studied, including monitoring therapeutic responses[15, 16], identification of specific genomic alterations to guide therapeutic selection [26], detection of minimal residual disease following surgical resection [13, 27], evaluation of mechanisms of resistance as a means to influence clinical decision making regarding a next line of treatment [10], and use to guide enrollment for patients to clinical trials based on potentially actionable mutations or amplifications[28]. Although several of the potential clinical indications for cfDNA assays require further clinical validation, there is inherent clinical utility in identification of actionable genomic alterations without a repeat invasive tissue biopsy when the initial tissue biopsy is insufficient for genotyping or uninformative, or at progression on matched therapy to identify actionable resistance mutations[23, 29]. For the test applied here, therapies matched to cfDNA-detected genomic alterations have demonstrated objective response rates comparable to tissue-based studies in advanced lung, breast and colon cancers in eight different publications [21, 30–35].

In our study, of the initial 128 patients, 50 had actionable genomic alterations identified by cfDNA profiling, and 15 were enrolled onto genotype-matched trials based on cfDNA results. Similarly low rates of enrollment based on tissue-genotyping results were reported by others, reflecting a limitation for such biomarker enrichment efforts. For example, from a cohort of 2,000 consecutive patients with advanced cancer who underwent testing on a genomic testing protocol, only a small minority (83 patients) in this large-scale testing using tissue genotyping were enrolled onto clinical trials targeting the alterations [36]. Indeed, the chance of a patient being enrolled into a trial depends on a variety of factors including assay turn-around time [17]. Availability of clinical trials differs not only across cancer types, but also according to a particular institution at any given time point. Although cancer physicians from tertiary-care cancer center varied considerably about the incorporation of genomic tests into practice[37], it has already been demonstrated that identifying specific molecular abnormalities and choosing therapy based on these abnormalities is relevant in both clinical trials[38] and clinical practice [39]. Since results from cfDNA assays can bring reliable information in a faster and noninvasive way, we believe it will be play an important role in this regard.

Although patients have been prospectively consented to be part of the ATTACC protocol (NCT01196130), the present study was observational in design and does have limitations. For instance, our finding that physicians perceived improvements in the quality of care and patients’ satisfaction with the use of cfDNA assays could have been biased by their preconceptions about cfDNA. Similarly, this survey was obtained from the perspective of the providers, and may not reflect the opinions and preferences of patients. Also, the data and analyses presented are exploratory, which can lead to bias in the interpretation of the findings. The level of concordance between FFPE tissue and cfDNA analyses are beyond the scope of the current work and will be addressed in a different publication.

In conclusion, our data suggest that the cfDNA targeted sequencing test evaluated here can provide clinically relevant information for potentially targetable mutations and amplifications. Subsequent to completion of our study, the cfDNA test has become comprehensive (i.e. inclusive of all four major types of alterations), which will likely become important as new genomic targets such as *RSPO3* and other fusions, or *ERBB2* (HER2) and *MET* gene copy number amplifications, may become potential targets on the near horizon [5, 40, 41]. Findings from sequencing of cfDNA can inform the provider of matched biomarker-based clinical trials while also improving the perceived quality of care. Turn-around time, utility and convenience, for providers and patients alike, appear to favor cfDNA over traditional sequencing of archival FFPE tissue. These data provide further impetus towards incorporation of this methodology into routine clinical care in the management of advanced solid tumors like metastatic colorectal cancer.

## Supporting information

S1 Fig Provider survey. (DOCX)

S2 Fig Number of genomic alterations detected by cfDNA analysis. cfDNA = cell-free DNA; Amplif = amplification.
(TIF)

S1 Table 54-gene NGS panel. (DOCX)
