# Supplementary material for: Clinical utility of circulating cell-free DNA in advanced colorectal cancer
Source: PLoS One. 2017 Aug 29;12(8):e0183949. doi: 10.1371/journal.pone.0183949 (PMC5574560; doi:10.1371/journal.pone.0183949)
Supplement: S1 Fig — (DOCX) [file pone.0183949.s001.docx]

### Supplemental Methods: Physician Utility Survey

1. Do you feel that the testing performed as part of the ATTACC program improved the quality of care that you could provide to the patient?
   1. Yes
   2. If No, why not?
2. Do you feel that the testing performed as part of the ATTACC program (including companion Clearinghouse and CMS400 protocols if performed) improved the patient’s satisfaction with the efforts to personalize experimental options?
   1. Yes
   2. If No, why not?
3. Do you feel that the plasma sequencing improved the quality of the care that you could provide to the patient?
   1. Yes
   2. If No, why not?
4. Do you feel that the plasma sequencing improved the patient’s satisfaction with the efforts to personalize experimental options?
   1. Yes
   2. If No, why not?
5. For this patient, what was the convenience of the plasma test results compared to tissue testing?
   1. Plasma testing was more convenient than tissue testing
   2. Tissue testing was more convenient than plasma testing
6. Were any of the results from the plasma sequencing “potentially actionable” in your estimation? (without regards to study availability)
   1. Yes, mutations were actionable
   2. Yes, amplifications were actionable
   3. Yes, both mutations and amplifications were actionable
   4. No, there were no findings or none of the findings were actionable
7. If Yes to #6, were you able to use the plasma sequencing results to identify specific potential clinical trials for your patient?
   1. Yes, at least one trial was identified
   2. No, I have reviewed clinical trials but have not found an option
   3. N/A, I have not yet reviewed
8. If Yes to #7, were you able to enroll your patient on the clinical trial?
   1. Yes, I already have or anticipate enrolling the patient
   2. No, there were no spots available
   3. No, the patient was not interested
   4. No, the patient did not meet eligibility criteria
9. Were there any “potentially actionable” mutations identified by sequencing of tumor tissue that were not identified with the plasma sequencing?
   1. Yes, additional ”potentially actionable” amplifications were present in the tissue
   2. Yes, additional ”potentially actionable” mutations were present in the tissue
   3. Yes, both additional ”potentially actionable” amplifications and mutations were present in the tissue
   4. No additional findings were present
10. Considering the goal of bringing molecular tumor characterization into the clinic to guide experimental therapy, which platform was the superior platform for this patient’s case?
    1. Sequencing of tumor tissue
    2. Sequencing of plasma

**Prespecified datapoints to be collected by study team:**

1. Were results available from the plasma analysis for this patient?
   1. Yes
   2. No, technically successful, but no mutations or amplifications were identified
   3. No, sample was not analyzed due to technical issues
2. Was tumor tissue available for this patient for concurrent testing?
   1. Yes, from a metastatic site within the past 3 months
   2. Yes, from a metastatic site, but not within the past 3 months
   3. Yes, from the primary tumor
   4. Tumor purity of tissue was too low for internal sequencing efforts
   5. No, the diagnostic tissue was exhausted or was not available
3. Was tumor testing done on a platform to allow identification of amplifications?
   1. Yes
   2. No
4. Were there findings present on the plasma sequencing that were not available by our internal sequencing efforts?
   1. Yes, additional ”potentially actionable” amplifications were present
   2. Yes, additional ”potentially actionable” mutations were present
   3. Yes, both additional ”potentially actionable” amplifications and mutations were present
   4. No additional findings were present
5. What was the speed of the test results compared to tissue testing?
   1. Plasma testing was faster than tissue testing
   2. Tissue testing was faster than plasma testing
