# Supplementary figures and images for: Clinical utility of circulating cell-free DNA in advanced colorectal cancer
Source: PLoS One. 2017 Aug 29;12(8):e0183949. doi: 10.1371/journal.pone.0183949 (PMC5574560; doi:10.1371/journal.pone.0183949)

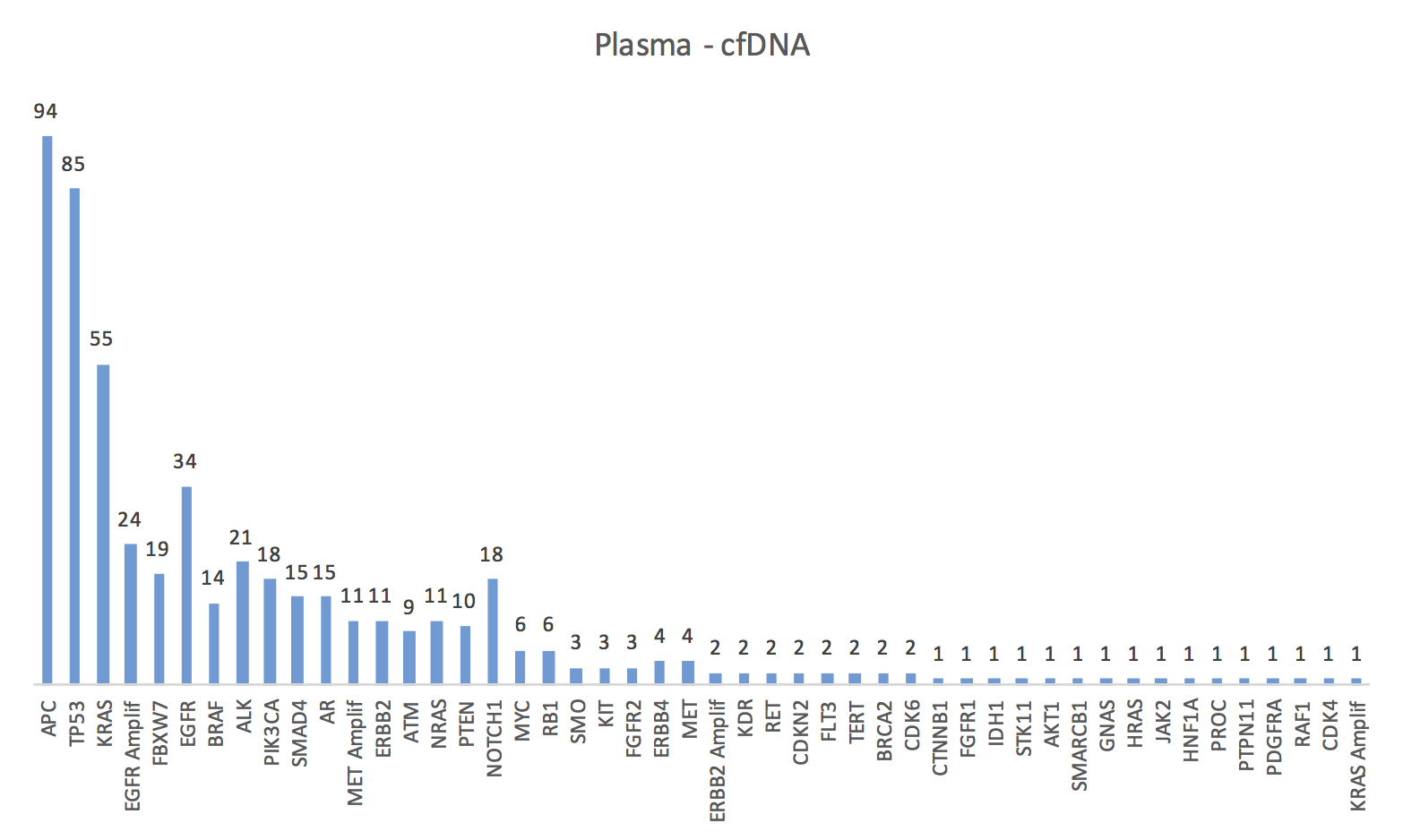

Supplement: S2 Fig — cfDNA = cell-free DNA; Amplif = amplification. (TIF) [file pone.0183949.s002.tif]
