# Supplementary material for: Clinical utility of circulating cell-free DNA in advanced colorectal cancer
Source: PLoS One. 2017 Aug 29;12(8):e0183949. doi: 10.1371/journal.pone.0183949 (PMC5574560; doi:10.1371/journal.pone.0183949)
Supplement: S1 Table — (DOCX) [file pone.0183949.s003.docx]

| *ABL1* | *AKT1* | *ALK* | *APC* | *AR* | *ATM* |
| --- | --- | --- | --- | --- | --- |
| *BRAF* | *CDH1* | *CDKN2A* | *CSF1R* | *CTBBB1* | *EGFR** |
| *ERBB2** | *ERBB4* | *EZH2* | *FBXW7* | *FGFR1* | *FGFR2* |
| *FGFR3* | *FLT3* | *GNA11* | *GNAQ* | *GNAS* | *HNF1A* |
| *HRAS* | *IDH1* | *IDH2* | *JAK2* | *JAK3* | *KDR* |
| *KIT* | *KRAS* | *MET** | *MLH1* | *MPL* | *MYC* |
| *NOTCH1* | *NPM1* | *NRAS* | *PDGFRA* | *PIK3CA* | *PTPN11* |
| *PTEN* | *PROC* | *RB1* | *RET* | *SMAD4* | *SMARCB1* |
| *SMO* | *SRC* | *STK11* | *TERT* | *TP53* | *VHL* |

*Copy number amplification of *EGFR*, *ERBB2* (HER2) and *MET* genes.

Supplemental Table 1 – Guardant360 54-Panel (June 2014 – January 2015)
